# Safety, efficacy and utility of methods of transferring adhesive and cohesive *Escherichia coli* cells to microplates to avoid aerosols

**DOI:** 10.12688/f1000research.5659.2

**Published:** 2015-01-14

**Authors:** Bryan Ericksen

**Affiliations:** 1 University of Maryland School of Medicine, Institute of Human Virology, 725 W. Lombard St., Baltimore, MD, USA

**Keywords:** Aerosols, Aerobiology, Quantitative Growth Kinetics, Environmental Monitoring

## Abstract

The virtual colony count (VCC) microbiological assay has been utilized for over a decade to measure the antimicrobial activity of peptides such as defensins and LL-37 against biosafety level (BSL)-1 and BSL-2 bacteria including *Escherichia coli*, *Staphylococcus aureus*, *Bacillus cereus*, and *Enterobacter aerogenes*.  In addition, a modified pipetting technique was presented in a 2011 study of defensin activity against the BSL-3 pathogen *Bacillus anthracis*.  Both studies were published in the journal Antimicrobial Agents and Chemotherapy.  Here I report that the method can also detect cross-contamination caused by aerosols utilizing the VCC method of data analysis by quantitative growth kinetics (QGK).  The QGK threshold time, or T _t_, equivalent to the cycle time C _t_ reported in 1996 by Heid et al., precisely identifies when wells were inoculated.

## Introduction

The virtual colony count (VCC) microbiological assay has been utilized for over a decade to measure the antimicrobial activity of peptides such as defensins ( Lehrer & Lu, 2012) and LL-37 ( Pazgier *et al.*, 2013). The initial VCC publication ( Ericksen, 2005) used two methods of transferring cells to microplates using a 20–200 µl multichannel pipettor: 22.2 µl added to 200 µl of media in calibration experiments and 50 µl added to 50 µl of solutions in phosphate buffer. Further experimentation has demonstrated that only the former method safely and effectively transfers cells to the intended wells, and the latter method can result in cross-contamination.

The reason for this difference is that adding cells suspended in 50 µl directly to a like volume caused unacceptable froth, bubbles and background turbidity that is incompatible with the VCC method of measuring growth kinetics by an increase in optical density using a 96-well plate in a plate reader. This problem, which affects optical density readings in turbidimetric assays, was initially solved by holding pipette tips just above the liquid but below the rims of the wells and adding cell suspensions as droplets. Accurately holding the multichannel pipettor within this narrow range seemed to require placing one’s eyes as close as possible to the 96-well plate, but further experiments using biosafety cabinets have proven that the method can be done by a well-trained operator looking through the glass. Assays conducted in 2012 and 2013 within a biosafety cabinet at the University of Maryland Baltimore (UMB) resulted in frequent cross-contamination of the 36 contamination control edge wells. Light microscopy revealed adhesive and cohesive clumps and biofilms formed by *Escherichia coli* ATCC 25922 and *Staphycococcus aureus* ATCC 29213. Changes in particle size distribution and adhesive properties due to clumping apparently resulted in increased aerosol formation, which made cross-contamination far more common than in the initial studies in 2003–2004 preceding the 2005 publication of VCC. Using this procedure for hazardous microorganisms outside a biosafety cabinet would pose a safety risk.

## Results

Growth kinetics optical density readings for Experiments 1 and 2 Dataset 120613 contains the raw Tecan output for Experiment 1. Only wells A11-H11 and A12-H12 are reported in this paper. Dataset 121813 contains the raw Tecan output for Experiment 2. Only wells A10-H10, A11-H11 and A12-H12 are reported in this paper. See the two .txt files for further information.

**Figure 1.**
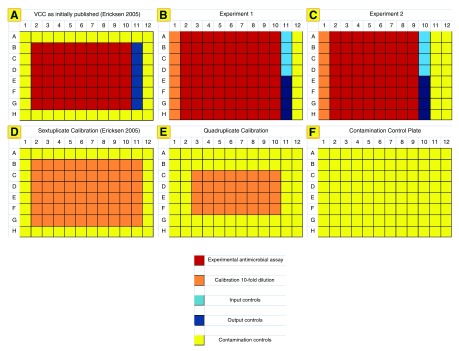
96-well plate configurations. Panels **B** and **C** depict contamination control wells on the right edge (columns 11–12) so that the eight-channel pipettor passes over them if when used by a right-handed operator. These wells could be moved to the left edge if the operator is left-handed.

The VCC plate configuration as initially published in 2005 ( Figure 1A) used the 36 wells around the edge of the 96-well plate (rows A and H and columns 1 and 12) as contamination control wells. Turbidity in these wells could have been the result of either environmental contamination or cross-contamination, but sampling wells over the course of many experiments revealed colony morphologies that were almost invariably consistent with the bacterial strain studied that day. Six alternating VCC experiments using *Escherichia coli* ATCC 25922 and *Staphylococcus aureus* confirmed this conclusion by producing colonies only consistent with the strain studied that day, not the strain studied in the previous experiment or an environmental isolate with a colony morphology matching neither strain.

Two hypotheses regarding the origin of cross-contamination were pursued: cells emanating from the pipette tips as they were passed directly over the contamination control wells or cells ejected up out of the wells as aerosols when the cell suspension was expelled. To distinguish between these possibilities, 13 experiments were conducted not with a single ring of 36 contamination control wells around the edge, but with an additional ring (columns 2 and 11 and rows B and G), totaling 64 uninoculated wells ( Figure 1E). In these experiments, quadruplicate 8-point 10-fold calibration dilutions were made by adding 22.2 µl beneath 200 µl of media, pipetting up and down 15 times, expelling tips, transferring 22.2 µl to the next column of four wells, etc. None of the 832 uninoculated wells turned turbid after overnight incubation at 37 degrees shaking in a Tecan Infinite M1000 plate reader, indicating a lack of cross-contamination or environmental contamination that is viable in rich media originating from the laboratory, reagents, operator or plate reader. Next, several VCC experiments were conducted using eight cross-contamination control wells in column 12 ( Figure 1B) with controls lacking antimicrobial agents in column 11 as described in the initial 2005 paper, during which all 24 cross-contamination control wells in column 12 turned turbid in all three experiments. Four changes were made to the procedure in an attempt to remove possible sources of contamination that may have caused cells to become more adhesive and cohesive, which in turn would have caused cross-contamination to become far more likely: 1. using a small HEPA-filtered air purifier, 2. replacing in-house deionized Milli-Q water with purchased molecular biology grade water, 3. replacing 2XMHB prepared and autoclaved in-house using reusable jars with Teknova 2X cation-adjusted MHB, and 4. filter-sterilizing phosphate buffers made near the portable air purifier, rather than autoclaving in reusable jars. After those changes, a 25 mL TSB culture grown as a biosensor simultaneously with the growth of the VCC seed culture no longer produced macroscopic clumps with diameters on the scale of millimeters. However, cross-contamination in VCC experiments persisted. In several of these experiments, a separate 96-well plate containing media only was interposed between the reagent reservoir containing the cell suspension and the experimental 96-well plate ( Figure 1F), and in no case did any well in these additional plates turn turbid. Had cells been transiently adhering to the outsides of the tips or trailing from the liquid held by capillary action at the openings of the tips, many or all of the 96 wells of the cross-contamination plates would have turned turbid, since all cross-contamination wells in column 12 on the right edges of experimental plates turned turbid. Therefore, contamination caused by passing the tips over these wells without expelling was ruled out. The next simplest explanation is that, while the plunger of the multichannel pipettor was depressed to deliver cells as droplets below the rims but above the liquid in the wells, the tips expelled viable aerosols that travelled in an upward trajectory and escaped the intended wells in such great numbers that the cross-contamination of adjacent wells was probable to the point of inevitability.

**Figure 2.**
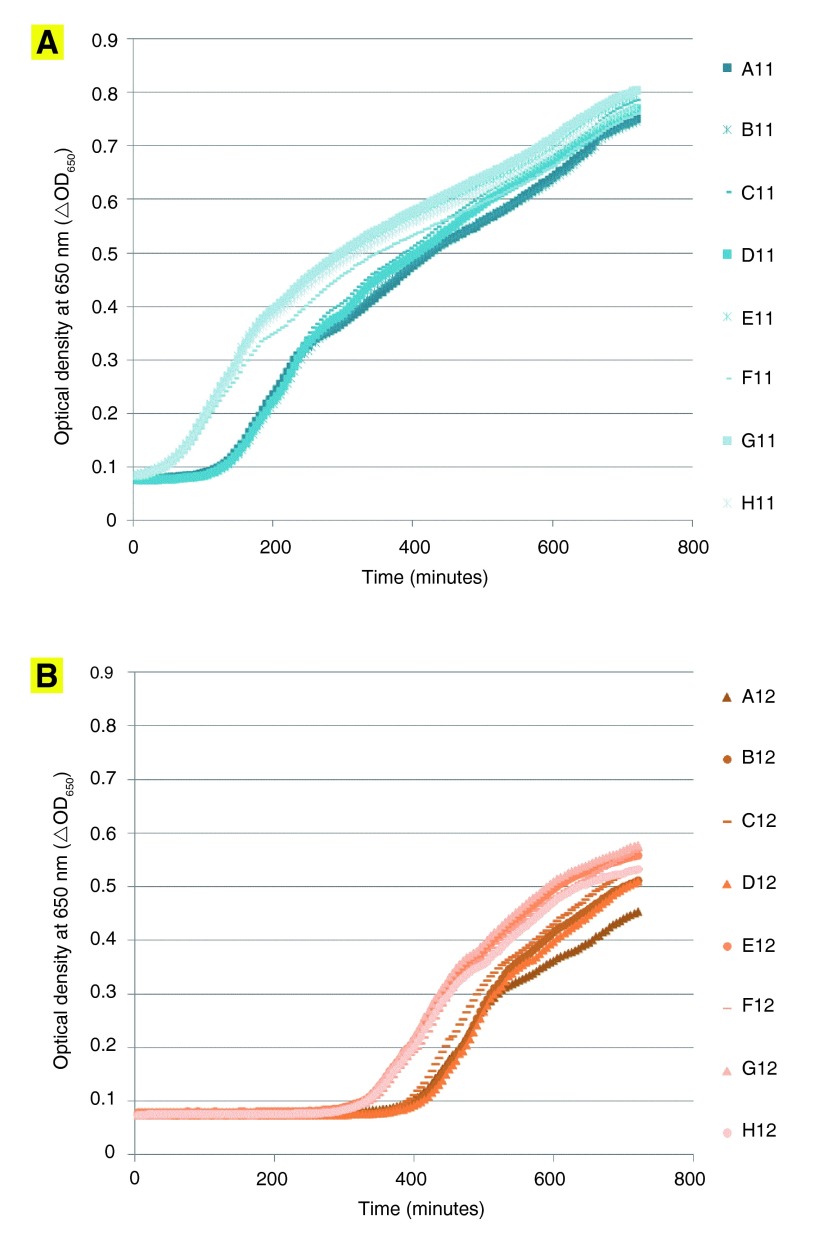
Uncorrected growth kinetics of columns 11 (panel **A**) and 12 (panel **B**) of the 96-well plate in Experiment 1. In these two columns (n=16), the threshold ΔOD _650_ value of 0.02 corresponded to a mean ± standard deviation uncorrected OD _650_ of 0.0989 ± 0.0043, which corresponds to a %RSD of 4.4. The line marked “0.1” is approximately at the position of the threshold ΔOD _650_ of 0.02.

In Experiment 1, configured as shown in Figure 1B, all eight wells in column 12 turned turbid and produced growth curves with the same growth rate and doubling times as the other growth curves on the same microplate ( Figure 2). Colony morphologies of samples from these wells also matched *E. coli* ATCC 25922. A comparison of threshold times indicated almost the same difference between input and output controls in columns 11 and 12 ( Table 1). There was a roughly 70-minute difference in input and output threshold times in the input and output control wells in Experiment 1, which agreed closely with another roughly 70-minute difference in the threshold times of the adjacent wells. Contamination caused by viable environmental strains would have been expected to produce widely varying threshold times, if not visible differences in the appearance of the turbid wells. Therefore, the 70-minute difference indicated that the cross-contamination occurred at the same time that cells were transferred.

**Table 1.** Experiment 1 (Dataset 120613) threshold time (T _t_) values.

		Columns	
		11	12
Rows	A	121.0	393.9
	B	124.3	398.8
	C	120.8	385.8
	D	122.2	403.7
	E	48.4	322.8
	F	50.4	333.0
	G	47.9	318.2
	H	48.2	325.4
	Mean, A-D	122.1	396.1
	Mean, E-H	48.7	324.9
	Mean, output minus Mean, input	73.3	71.2

A11-D11 are the “input” control wells and E11-H11 are “output” control wells. Cells were added to those two wells two hours apart, resulting in a 73.3 minute difference in T _t_ values. Cross-contaminated wells gave a corresponding T _t_ difference of 71.2 minutes, indicating that A12-D12 were inoculated as cells were being expelled over A11-D11, and E12-H12 were inoculated as cells were being expelled over E11-H11.

**Figure 3.**
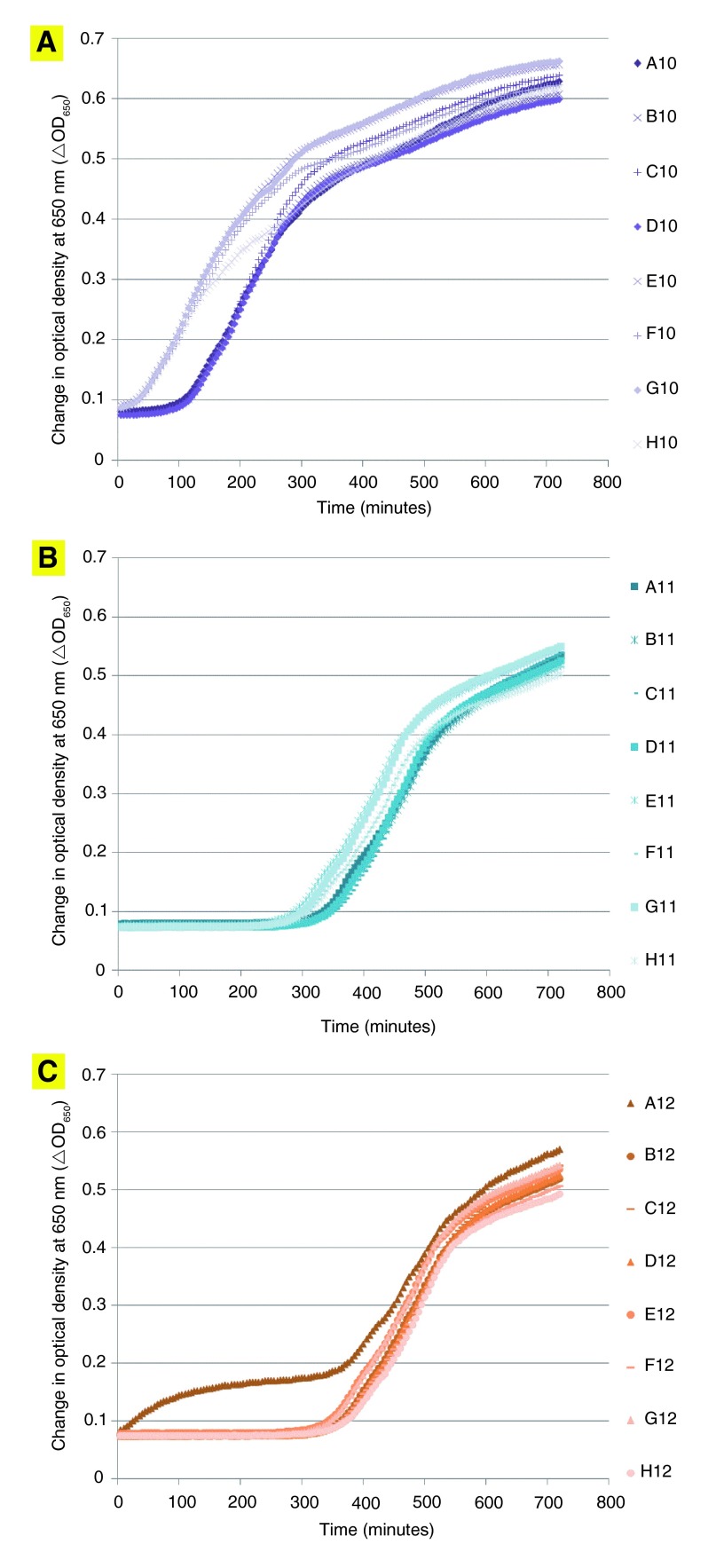
Uncorrected growth kinetics of columns 10 (panel **A**), 11 (panel **B**) and 12 (panel **C**) of the 96-well plate in Experiment 2. In these three columns excluding well A12 (n=23), the threshold ΔOD _650_ value of 0.02 corresponded to a mean ± standard deviation uncorrected OD _650_ of 0.0988 ± 0.0053, which corresponds to a %RSD of 5.4. The biphasic curve in well A12 was unique among the 96 wells analyzed in this assay, and is caused by an initial phase of optical density increase caused by condensation on the lid followed by a second phase caused by increased turbidity due to cell growth within the well.

In Experiment 2, configured as shown in Figure 1C, the threshold times again reflected a roughly 70-minute difference between input and output controls. ( Figure 3 and Table 2) However, this difference was not reflected in threshold times of the cells growing in column 12, suggesting that the contamination of those wells was the result of either a second contamination event unrelated to the timing of the transfer of cells into the wells in column 10 or a lower inoculum in each well. The only reasonable explanation of this agreement in threshold time differences between columns 10 and 11 and the far larger T _t_ values resulting from column 12 is that cross-contamination occurred while cells were expelled, and the aerosols thus formed travelled to the adjacent wells but not the intervening 96 contamination control wells in the contamination control plate, none of which turned turbid after overnight incubation at 37 degrees. These results indicate that 96-well plates and threshold times are useful for detecting contamination, and that cross-contamination occurs in experiments where cells are added as droplets from above.

**Table 2.** Experiment 2 (Dataset 121813) threshold time (T _t_) values.

		Columns		
		10	11	12
Rows	A	106.7	332.3	30.6
	B	109.2	335.5	354.9
	C	108.2	341.9	368.7
	D	109.8	335.4	358.3
	E	40.4	282.4	340.5
	F	41.0	299.1	357.6
	G	39.8	290.5	340.9
	H	39.6	303.5	364.3
	Mean, A-D*	108.5	336.3	360.6
	Mean, E-H	40.2	293.9	350.8
	Mean, B-H			355.0
	Mean, output minus Mean, input	68.2	42.4	
	output-input minus cross- output-input		25.8	
	Mean, B12-H12 minus mean A11-D11		18.7	

A10-D10 are the “input” control wells and E10-H10 are “output” control wells. Cells were added to those two wells two hours apart, resulting in a 68.2 minute difference in T _t_ values. Cross-contaminated wells gave a corresponding T _t_ difference of 42.4 minutes. The difference between these two values, 25.8 minutes, could be accounted for by the growth of additional cells added in a second contamination event reflected by wells B12-H12 T _t_ values that also caused media in the reservoir to turn turbid when collected and incubated overnight. Thus, T _t_ values detect cross-contamination in adjacent wells and can distinguish between separate contamination events.

## Discussion

The method of enumeration of cells in a VCC assay is confounded if the cells form clumps, because that clumping and biofilm formation affects optical density readings. Other experiments using tryptic soy broth (TSB) media rather than the Mueller-Hinton Broth (MHB) media chosen for the initial VCC publication in 2005 revealed macroscopic clumps and biofilms visible to the unaided eye. In addition, microscopic clumps were revealed by light microscopy in both TSB and MHB. Cohesion, adhesion, clumps and biofilms affect not only threshold times but also the particle size distribution of the cell suspension and the degree of adhesion as the cells are expelled through the pipette tips. Therefore, both cells adhering to surfaces and cohesive clumps suspended in solution formed by cells adhering to each other but not surfaces could affect the physical properties of the liquid as it is transformed to an emulsion that generates aerosols. Cross-contamination was far more common in the experiments I conducted in 2012–2013 compared to experiments I conducted in 2003–2004 in an adjacent room, suggesting that some change in environmental factors between those times or locations caused greater cell clumping and adhesion, which in turn greatly increased the probability that a cross-contamination control well would become turbid. Environmental factors (EFs) clearly play a role in this phenomenon. Three categories of EFs evidently affected the two experiments reported here: adhesive, bubble-forming, and clumping. Adhesive environmental factors (AEFs) could explain the presence of plate reader artifacts manifested as condensation on the upper or lower surfaces of the polystyrene 96-well plate lids. An AEF landing near the center of a well with a residence time sufficient to act as a condensation nucleus could be responsible for the condensation kinetics exhibited in well A12 of Experiment 2. ( Figure 3C) Many compounds and particles can serve as condensation nuclei, including molecules as small as dimethyl sulfate, a condensation nucleus produced by cyanobacteria that influences cloud formation and global weather patterns. ( Charlson *et al.*, 1987) Bubble-forming EFs (BEFs) transmitted through the air might explain the froth and turbidity that initially necessitated the rejection of the direct addition of a 50 µL cell suspension to 50 µL of phosphate buffer. In addition, BEFs were evident when electronic multichannel pipettors were tested in 2003, resulting in large bubbles within the pipette tips. Even returning to a manual eight-channel pipettor, bubbles often entered one or more of the tips, requiring the liquid to be expelled. These observations are probably influenced by the peculiarities of the laboratory environment at IHV. A third type of EF, clumping EFs (CEFs), caused cells to precipitate and form persistent clumps and biofilms on the bases of the wells. CEFs also caused cells to form microscopic rings and circles observed using light microscopy by adding lactophenol cotton blue to Gram-stained slides, and macroscopic clumps in 125 mL filter flasks and cuvettes. It is postulated that one or more clumping environmental factor (CEF) is responsible for the change in cross-contamination frequency between 2003 and 2014 and a 23-fold fluctuation in virtual lethal dose values reported by the HNP1 positive controls of the assay in *E. coli* ATCC 25922 VCC experiments in 2013.

In 2003–2004, most VCC experiments generated no turbid cross-contaminated wells using six strains of four bacterial species: *E. coli, S. aureus, Enterobacter aerogenes* and *Bacillus subtilis.* Among experiments that exhibited cross-contamination, one turbid well out of the 36 cross-contamination edge wells was the most likely result. On rare occasion, two wells became turbid, and one experiment produced 12 turbid wells. Because contamination appeared to be rare in these experiments, the pipetting solution of holding tips above the liquid in the wells when adding cells was judged to be acceptable, and no further investigation was conducted at that time. In retrospect, however, any turbidity in contamination control wells should be investigated further. Nonzero cross-contamination tallies probably indicated that EFs were present and affected experimental results from the beginning, even though the lower frequency of contamination initially suggests that the influence of EFs increased between 2003 and 2014.

In 2011, a modified VCC procedure ( Welkos *et al.*, 2011) was published for use with the BSL-3 pathogen *Bacillus anthracis*, ** based on the procedure originally developed at UCLA in the laboratory of Robert I. Lehrer. The 50 µl cell transfer step mentioned in the 2005 VCC publication and used at the University of Maryland was replaced with the addition of cells suspended in a smaller volume of liquid, 10 µl, added to 90 µl of buffer. This procedure, similar to the calibration experiments detailed in the original VCC publication ( Figure 1D), did not generate unacceptable turbidity when cell suspensions were added with the tips placed at the bases of the wells beneath the buffer when it was tested in 2013 in the IHV building at UMB. Adding cell suspensions under liquid apparently greatly reduces the probability of aerosol formation, which is of concern not only for safety reasons, but also because the aerosol cloud within the well can alter experimental results by generating cells that adhere to the sides of the well during the exposure to the antimicrobial agent, then drop down to inoculate the outgrowth media after the antimicrobial peptides have been neutralized by broth during 12 hours of vigorous shaking within the plate reader. VCC users are cautioned to use the 2011 procedure, not the 2005 procedure, to add experimental cell suspensions. Following the 2005 procedure to add *Staphylococcus aureus* cell suspensions in droplets above the liquid in the wells rather than injecting the cell suspension beneath the liquid in the wells could expose the eyes to aerosols containing a biosafety level 2 pathogen that could cause blepharitis, corneal stromal microabscess, stromal edema, uveitis, ocular necrotizing fascitis, and blindness. ( Boto-de-Los-Bueis *et al.*, 2014; Shield *et al.*, 2013) Biosafety level 2 precautions such as those recommended by the Centers for Disease Control in *Biosafety in Microbiological and Biomedical Laboratories, 5 ^th^ Edition* ( Miller *et al.*, 2012) should be taken for any study of *Stapylococcus aureus*, including the safer 2011 VCC procedure.

Adding cells beneath liquid results in more thorough mixing than adding cells above it, especially when the additional detail of pipetting the liquid up and down with the tips placed in cross-sectional corners is employed. However, thorough mixing is a greater concern for the 10-fold dilutions of the calibration experiment than for experimental assays, because it must occur in a far shorter period of time. Pipetting to mix 15 times was employed for each column of wells to produce 10-fold dilutions in order to set up the plate quickly. Cells were added at room temperature, not on ice or at 37°C, and it was desirable to limit the duration of this temperature excursion. In addition, diluting cells quickly minimized the risk of contamination by limiting exposure to ambient air with the lid off the plate. Rapid mixing might be less important for the experimental portion of the assay, when cells are not diluted in the 96-well plate. The overall duration of the two-hour incubation period in the presence of antimicrobial peptides would greatly overshadow whatever initial diffusion time might be necessary to achieve a homogeneous suspension.

**Figure 4.**
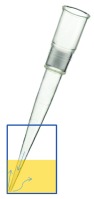
Position of pipette tips beneath liquid and in contact with the cross-sectional corners of the wells. Arrows indicate the flow of a cell suspension as the liquid is expelled, generating mild shear to disperse clumps and maximize mixing efficacy.

On the other hand, pipetting up and down beneath liquid is undoubtedly an improvement. The mild shear resulting from the proximity of the well surfaces to the tip opening ( Figure 4) would not only tend to disperse cohesive clumps, it would also yield a more homogeneously mixed suspension of single cells. Adding cells as droplets from above utilizes only the shaking of the plate within the plate reader to mix the cells with the buffer underneath. This shaking occurred in a linear fashion for about 15 seconds initially, then every five minutes for three seconds duration throughout both the two-hour and twelve-hour incubation steps of the assay in experiments at UMB using a Molecular Devices Vmax plate reader in a 37°C warm room between 2003 and November, 2011. Thereafter, the assay was adapted for a temperature-controlled Tecan Infinite M1000 plate reader, which allows for additional shaking options including either orbital or linear shaking and near-continuous shaking between readings. Sampling the volume of wells at various locations after the addition of cells, followed by plating and colony counting to compare the efficacy of various methods of mixing, might clarify this question further. It should be noted that the UCLA experiments published in 2011 utilized a Molecular Devices Spectramax plate reader, which is temperature-controlled with shaking features similar to the Vmax. Bacterial growth kinetics might vary somewhat in these three plate readers due to changes in aeration and temperature control, and airflow might in turn affect the magnitude of the influence of EFs on experimental results.

These results highlight an advantage of using the VCC data analysis procedure of enumerating cells ( Brewster, 2003), termed quantitative growth kinetics (QGK) by analogy to quantitative polymerase chain reaction (QPCR). ( Heid *et al.*, 1996) QGK and QPCR use a mathematically identical procedure for quantifying the initial number of cells or amplicons that were present at the start of the assay. The QGK threshold time T _t_ is equivalent to the PCR cycle time C _t_. Calculating T _t_ values in the two experiments reported here unequivocally identified the time of the contamination event, gave quantitative batch culture growth kinetic data that suggested that the contamination was cross-contamination, and distinguished between two inocula. These features of QGK would greatly improve the quality of environmental monitoring data when used to detect contamination by aerosols or ambient viable microorganisms compared to turbidity measurements in the absence of a plate reader or observing the appearance of colonies on agar plates, neither of which provides kinetic data.

Finally, it should be emphasized that the simple improvement of adding cells beneath liquid simultaneously achieves two useful changes at once, reducing the probability that cells inoculate wells other than the ones intended while simultaneously also limiting the probability that cells escape the 96-well plate entirely. Although the reason why the addition of 50 µl of cells beneath 50 µl of liquid was unacceptable in VCC experiments stemmed from the turbidimetric nature of the assay, this method of preventing cross-contamination is far from trivial or confined to VCC assays. It teaches a technical lesson limited not just to environments where airborne AEFs, BEFs and CEFs are present, but broadly applicable to all experiments where microbes are transferred using pipette tips, thereby potentially improving the usefulness of a wide range of laboratory procedures that might otherwise generate aerosols. Any change in a procedure that improves its safety and efficacy also improves its utility *ad oculos*.

## Materials and methods

VCC assays were conducted as described ( Ericksen, 2005) and modified ( Zhao, 2013). Twice-concentrated cation-adjusted Meuller Hinton Broth was purchased from Teknova, Inc. Phosphate buffers were made using Sigma monobasic and dibasic sodium phosphate dissolved in molecular biology grade water or equivalent purchased from multiple sources. Rainin GreenPak LTS 200 µl filter tips were used with an eight-channel 20–200 µl pipettor. Costar 3595 96-well plates were analyzed in a Tecan Infinite M1000 plate reader at 37°C for two hours before media addition, then 12 hours afterward.

Two experiments were conducted using *Escherichia coli* ATCC 25922. In Experiment 1, four each of “input” and “output” controls were placed in column 11 of the 96-well plate, with eight cross-contamination control wells in column 12. Wells E11-H11 contained controls in wells added at the time the cells were exposed to antimicrobial agents, termed the “output” controls, and equivalent to the controls mentioned in the initial 2005 publication. In addition, wells A11-D11 contained identical controls that had been stored on ice during the two-hour exposure to antimicrobial agents in phosphate buffer, termed the “input” controls because their T _t_ values represent the concentration of cells that were present when they were put into the assay at the start of the two-hour incubation. Since the antimicrobial assay is beyond the scope of this report, which focuses only on aerosol cross-contamination, columns 1–10 and the antimicrobial agents therein will not be discussed here.

**Figure 5.**
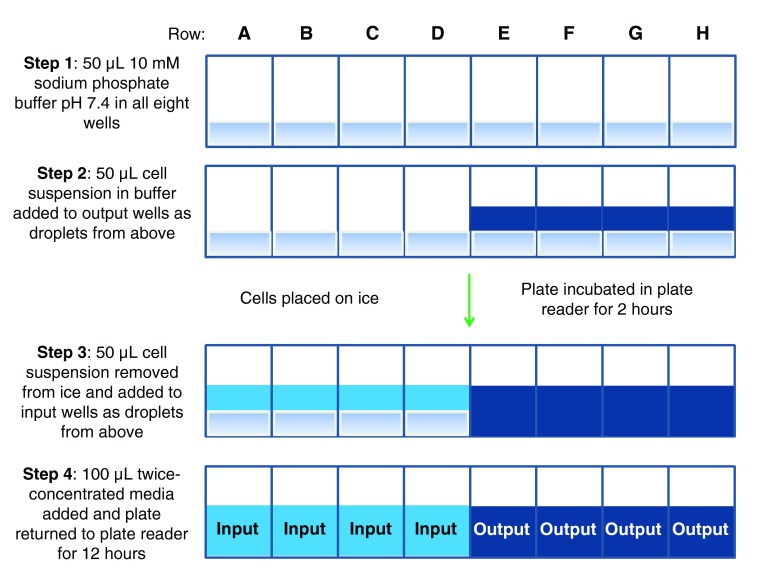
Cross-sectional depiction of the procedure for the addition of input and output control *E. coli* cell suspensions in phosphate buffer to column 11 of Experiment 1 and column 10 of Experiment 2.

**Figure 6.**
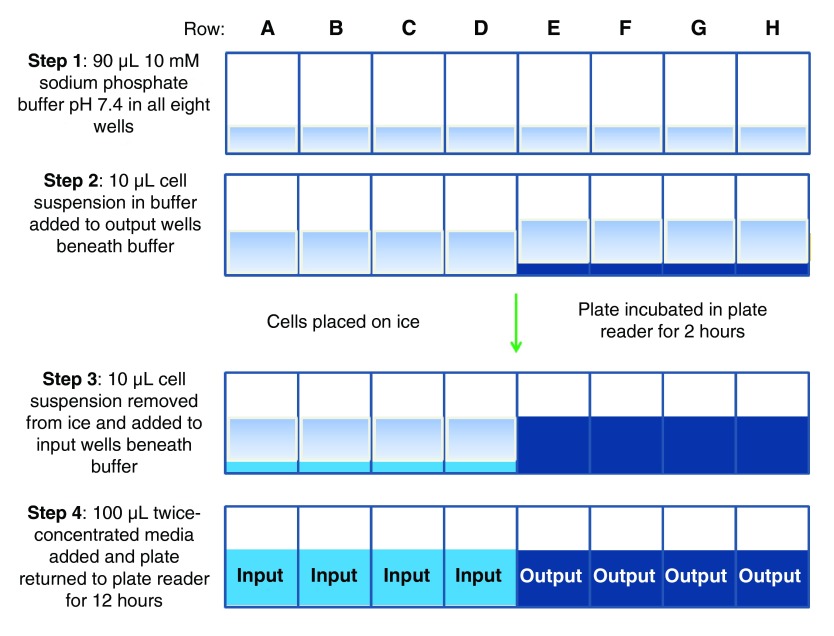
Safer, more effective, and more useful adaptation of the method of transferring cells depicted in Figure 5. A five-fold more concentrated inoculum is added in one-fifth the volume beneath buffer rather than as droplets added from above.

Next, in Experiment 2, the controls lacking antimicrobial agents were moved from column 11 to column 10, and columns 11 and 12 contained 16 uninoculated contamination control wells. Wells E10-H10 contained output controls and wells A10-D10 contained input controls ( Figure 5). These controls are designed such that comparing the difference in threshold times between the input and output controls, relating that difference to the calibration curve elsewhere on the same 96-well plate, and assuming that adhesion or cohesion and lag phases in exponential growth were the same for all cells, the growth of the cells during the two hour incubation on the plate could be quantified. Enumerating the change in cell concentration during that step would allow the calculation of the difference in virtual survival values that would correspond to bacteriostatic activity. Figure 6 depicts the improved methodology, requiring a fivefold more concentrated cell inoculum in buffer added in one-tenth, rather than one-half, of the 100 µL total volume of the 2-hour incubation step. This method has not been tested, and it is unknown whether the additional step of pipetting up and down 15 times to mix, as depicted in Figure 4, would be necessary to ensure proper mixing. The volumes shown in Figure 6, rather than Figure 5, used for the addition of cells in the red portions of the 96-well antimicrobial assays as designed in Figure 1, would lessen the probability of cross contamination.

## Data availability

F1000Research: Dataset 1. Growth kinetics optical density readings for Experiments 1 and 2, 10.5256/f1000research.5659.d38055 ( Ericksen, 2014).
