# Supplementary material for: Safety, efficacy and utility of methods of transferring adhesive and cohesive Escherichia coli cells to microplates to avoid aerosols
Source: F1000Res. 2015 Jan 14;3:267. Originally published 2014 Nov 6. [Version 2] doi: 10.12688/f1000research.5659.2 (PMC4309163; doi:10.12688/f1000research.5659.2)
Supplement: Peer review history

# Peer review: Referee response for version 2

**DOI:** 10.5256/f1000research.6437.r7472

**Published:** 2015-01-26

**Authors:** Dipshikha Chakravortty

**Affiliations:** 1 Department of Microbiology and Cell Biology, Indian Institute of Science, Bangalore, India

In light of the revisions and comments from the authors, I am happy to approve this article for indexing.

I have read this submission. I believe that I have an appropriate level of expertise to confirm that it is of an acceptable scientific standard.

# Peer review: Referee response for version 1

**DOI:** 10.5256/f1000research.6043.r6656

**Published:** 2014-11-27

**Authors:** Lynn Silver

**Affiliations:** 1 LL Silver Consulting LLC, Springfield, NJ, USA

The Virtual Colony Count was devised in the author’s laboratory and has been in use for over ten years.  It seems that this would be a very useful method for many applications as it can obviate the need for actual colony counting while still providing information about viability [although not “cfu” per se]. It would be useful to better introduce the system with a short explanation of the method itself so that new readers can see its potential benefits.

This paper is a careful demonstration of the source of contamination – the production of aerosols – which have occurred when, as in the original method, 50 microliters of cell suspension is delivered above the surface of  50 microliters of test compound in medium (although this was not problematic during the initial work).  Not surprisingly, contamination, and by deduction, aerosols were greatly reduced when a smaller volume, 22.2 microliters, was pipetted below the surface of 200 microliters of medium.  Is it known if the latter procedure leads to better initial mixing than the former, as might be expected? A set of 13 preliminary experiments was carried out to generate hypotheses about the origin of contamination seen in medium control wells, as outlined in the introduction.  It might be useful to show schematics of the plate formats used since following the details in the introduction is a little confusing. The same is true for the two final experiments that were performed to test the ultimate hypothesis. Also the terms input and output controls could be defined better. The difference between the 2012-2013 and 2003-2004 results is ascribed to “one or more clumping environmental factors (CEF)”.  First, why abbreviate the name to CEF as it is only used twice in the paper? Second, is it possible that differences could be due to changes in the labware used, such as pipette tips, pipettors, microtiter plates?  Even when purchased from the same vendor and ostensibly the same over time, it is possible that slight manufacturing changes could affect the results. For example, the 2013 set up might lead to less controllable expulsion of liquid.  The experiments are well planned and the work is carefully done.

I have read this submission. I believe that I have an appropriate level of expertise to confirm that it is of an acceptable scientific standard.

# Peer review: Referee response for version 1

**DOI:** 10.5256/f1000research.6043.r6659

**Published:** 2014-11-26

**Authors:** Dipshikha Chakravortty

**Affiliations:** 1 Department of Microbiology and Cell Biology, Indian Institute of Science, Bangalore, India

The manuscript titled "Safety, efficacy and utility of methods of transferring adhesive and cohesive Escherichia coli cells to microplates to avoid aerosols" by Bryan Ericksen reports a safer method for transferring  certain strains of E.coli. Though this report may generate some interest  the E.coli community, this paper will lack the diverse readership. Being a very focused Method paper, it is good to seek journals which reports methods.  As the load of microorganisms are very different in different countries, to adapt this technique for any lab will not be possible. It is good to have a lab standard using this technique and it will be restricted to that lab. Finally this entire concept is very qualitative.

I have read this submission. I believe that I have an appropriate level of expertise to state that I do not consider it to be of an acceptable scientific standard, for reasons outlined above.

# Response

**Published:** 2014-11-26

**Authors:** Bryan Ericksen

**Affiliations:** University of Maryland, USA

Dear Dr. ​Chakravortty,

Thank you for taking the time to read the paper and submit your review.  I am not sure what you mean by "load of microorganisms" or why you say these methods could not be adapted for other laboratories, but your comment regarding QGK and VCC lab-to-lab variability is well-taken, which would be an excellent topic for further study if the identical experiment were to be repeated in many laboratory environments around the world. That is one reason why encouraging safe pipetting techniques is so important.  Although I only presented *E. coli* data using a biosafety level 1 (BSL-1) strain in the figures and tables, the consequences of aerosol transmssion are perhaps greater for more dangerous microorganisms such as the BSL-2 pathogen *Staphylococcus aureus*, which was mentioned in the text.  QGK generally, and the VCC method specifically, could potentially apply to a wide variety of other bacteria, as has already been demonstrated.  The references mentioned in this work include the results of experiments performed using *Enterobacter aerogenes, Bacillus cereus*, and *Bacillus anthracis*.

However, your comment regarding the qualitative nature of threshold times is rather baffling.  It is precisely the quantitative, rather than qualitative, nature of QGK that permits its use to determine precisely when a turbid well was inoculated and thereby confirm aerosol transmission at the time cells were expelled as the mechanism of cross-contamination.  I would encourage a more careful reading of the paper and tables 1 and 2, which present quantitative, rather than qualitative, results.

Finally, I should explain why this methodological paper is so narrowly focused, as you noticed. Its genesis was an erratum to the original VCC paper (cited here as Ericksen 2005).  Here is the erratum text verbatim, which contains a critical methodological detail:

"The virtual colony count (VCC) microbiological assay has been utilized for over a decade to measure the antimicrobial activity of peptides such as defensins.  The initial VCC publication used two methods of transferring cells to microplates using a 20-200 microliter multichannel pipettor: 22.2 microliters added to 200 microliters of media in calibration experiments and 50 microliters added to 50 microliters of solutions in phosphate buffer.  Further experimentation has demonstrated that only the former method safely and effectively transfers cells to the intended wells, and the latter method can result in cross-contamination. 

The reason for this difference is that adding cells suspended in 50 microliters directly to a like volume causes unacceptable froth, bubbles and background turbidity that is incompatible with the VCC method of measuring growth kinetics by an increase in optical density using a 96-well plate in a plate reader.  This problem, unique to turbidimetric assays, was initially solved by holding pipette tips just above the liquid but below the rims of the wells and adding cell suspensions as droplets.  Assays conducted in 2012 and 2013 within a biosafety cabinet at the University of Maryland Baltimore (UMB) resulted in frequent cross-contamination of the 36 contamination control edge wells.  Light microscopy revealed adhesive and cohesive clumps and biofilms formed by *Escherichia coli* ATCC 25922 and *Staphycococcus aureus *ATCC 29213.  Changes in particle size distribution and adhesive properties due to clumping apparently resulted in increased aerosol formation, which made cross-contamination far more common than in the initial studies in 2003-2004 preceding the 2005 publication of VCC.  Using this procedure for hazardous microorganisms outside a biosafety cabinet would pose a safety risk.

In 2011, a modified VCC procedure was published for use with the BSL-3 pathogen *Bacillus anthracis*, **based on the procedure originally developed at UCLA in the laboratory of Robert I. Lehrer.  The 50 microliter cell transfer step was replaced with the addition of cells suspended in a smaller volume of liquid, 10 microliters, added to 90 microliters of buffer.  This procedure, similar to the calibration experiments detailed in the original VCC publication, did not generate unacceptable turbidity when cell suspensions were added with the tips placed at the bases of the wells beneath the buffer.  Adding cell suspensions under liquid greatly reduces the probability of cross-contamination., which is of concern not only for safety reasons, but also because the aerosol cloud within the well can alter experimental results by generating cells that adhere to the sides of the well during the exposure to the antimicrobial agent, then drop down to inoculate the outgrowth media after the antimicrobial peptides have been neutralized by broth during 12 hours of vigorous shaking within the plate reader.  VCC users are cautioned to use the 2011 procedure, not the 2005 procedure, to add experimental cell suspensions."

All four authors of the 2005 paper agreed to the above erratum text earlier this year.  In rejecting the erratum, the journal suggested conducting further experiments and submitting a separate paper, which I can no longer do, since I am no longer employed by the lab.  However, in previously conducted experiments I did not observe unacceptable froth and turbidity when I added a small volume of *E. coli *cells beneath liquid following the 2011 VCC method, and I had already proven that the 2005 VCC method resulted in cross-contamination due to aerosol formation based on experiments I conducted in 2013. Therefore, I wrote a draft of this paper and submitted it to Antimicrobial Agents and Chemotherapy, on the principle that errors reported in a given journal should be corrected in the same journal. Predictably, the paper was also rejected as too narrow.  I have served as a reviewer for the journal, and I would have agreed had I not known about the rejection of the erratum.  I appreciate the opportunity given to me by *F1000Research *to correct the literature in a separate peer-reviewed forum, which will have to suffice in place of an erratum to the original paper, and as a fortunate by-product of this process I have also had the opportunity to present a novel way of quantifying cross-contamination.  I believe aerosol transmission could explain some nosocomial infections and affect the experimental results of other assays where liquid is transferred, so I hope this modest paper will benefit the fields of assay development, bacteriology and aerobiology in some small way.

Sincerely,

Bryan Ericksen, Ph.D.
